# Gas Chromatography-Mass Spectrometry for Metabolite Profiling of Japanese Black Cattle Naturally Contaminated with Zearalenone and Sterigmatocystin

**DOI:** 10.3390/toxins9100294

**Published:** 2017-09-21

**Authors:** Katsuki Toda, Emiko Kokushi, Seiichi Uno, Ayaka Shiiba, Hiroshi Hasunuma, Yasuo Fushimi, Missaka P. B. Wijayagunawardane, Chunhua Zhang, Osamu Yamato, Masayasu Taniguchi, Johanna Fink-Gremmels, Mitsuhiro Takagi

**Affiliations:** 1 United Graduate School of Veterinary Sciences, Yamaguchi University, Yamaguchi 753-8515, Japan; t1643eson@yahoo.co.jp (K.T.); shiibaayakahime@yahoo.co.jp (A.S.); osam@vet.kagoshima-u.ac.jp (O.Y.); masa0810@yamaguchi-u.ac.jp (M.T.); 2 Laboratory of Theriogenology, Joint Faculty of Veterinary Medicine, Yamaguchi University, Yamaguchi 753-8515, Japan; 3 Faculty of Fisheries, Kagoshima University, Kagoshima 890-0056, Japan; kokushi@fish.kagoshima-u.ac.jp (E.K.); uno@fish.kagoshima-u.ac.jp (S.U.); 4 Shepherd Central Livestock Clinic, Kagoshima 899-1611, Japan; hasu@fa3.so-net.ne.jp (H.H.); yasuo243@gmail.com (Y.F.); 5 Department of Animal Science, University of Peradeniya, Peradeniya 20400, Sri Lanka; missakaw@pdn.ac.lk; 6 MILS International, Kanazawa 920-0222, Japan; mils@yacht.ocn.ne.jp; 7 Joint Faculty of Veterinary Medicine, Kagoshima University, Kagoshima 890-0062, Japan; 8 Faculty of Veterinary Medicine, Utrecht University, Yalelaan 104, The Netherlands; J.Fink@uu.nl

**Keywords:** cattle, GC/MS, metabolomics, sterigmatocystin, urine, zearalenone

## Abstract

The objective of this study was to evaluate the metabolic profile of cattle fed with or without zearalenone (ZEN) and sterigmatocystin (STC)-contaminated diets using a gas chromatography-mass spectrometry metabolomics approach. Urinary samples were collected from individual animals (*n* = 6 per herd) from fattening female Japanese Black (JB) cattle herds (23 months old, 550–600 kg). Herd 1 had persistently high urinary ZEN and STC concentrations due to the presence of contaminated rice straw. Herd 2, the second female JB fattening herd (23 months old, 550–600 kg), received the same dietary feed as Herd 1, with non-contaminated rice straw. Urine samples were collected from Herd 1, two weeks after the contaminated rice straw was replaced with uncontaminated rice straw (Herd 1N). Identified metabolites were subjected to principal component analysis (PCA) and ANOVA. The PCA revealed that the effects on cattle metabolites depended on ZEN and STC concentrations. The contamination of cattle feed with multiple mycotoxins may alter systemic metabolic processes, including metabolites associated with ATP generation, amino acids, glycine-conjugates, organic acids, and purine bases. The results obtained from Herd 1N indicate that a two-week remedy period was not sufficient to improve the levels of urinary metabolites, suggesting that chronic contamination with mycotoxins may have long-term harmful effects on the systemic metabolism of cattle.

## 1. Introduction

Contamination of agricultural commodities with mycotoxins, which are secondary metabolites of fungi, is a major problem in agriculture and livestock production worldwide [1]. The consumption of mycotoxin-contaminated products is generally believed to cause acute and chronic effects in humans and animals; thus, the contamination of food, feed, and ingredients with mycotoxins presents significant health risks [1,2]. In cattle production, several in vivo reports on the acute and chronic effects of mycotoxicosis have been published. Those reports are mainly based on clinical symptoms observed in field studies or following the experimental administration of mycotoxin to cattle, occasionally with concomitant biochemical analysis. However, risk evaluations concerning the metabolic status of cattle exposed to mycotoxin contamination are lacking [3]. 

Recently, we reported the presence of mycotoxin contamination in the dietary feed (rice straw) of a cattle herd by demonstrating the presence of zearalenone (ZEN) and sterigmatocystin (STC), which are produced by two groups of fungi, *Fusarium* spp. and *Aspergillus* spp., and their secondary metabolites in urine using our established liquid chromatography-tandem mass spectrometry (LC-MS/MS) monitoring technique [4,5]. Moreover, we suggested that monitoring ZEN and STC levels in urine is a practical and useful way of evaluating the contamination status of a cattle herd and assessing the efficiency of mycotoxin adsorbent, which is supplemented in dietary feeds to impair the intestinal adsorption of mycotoxins [3,4,6].

Because organisms must maintain homeostasis, metabolic profiles vary continuously with changes in their physical condition. Metabolomics can provide a quantitative description of endogenous metabolites of low molecular mass present in biological samples such as urine, plasma, tissue, and organs, or in the whole body of an organism [7,8]. Metabolomics can, therefore, provide information about the metabolic processes occurring in an organism, and has proven to be highly sensitive at evaluating the effects of disease [9], toxicants [10], and other stressors [11]. Recently, there have been reports of using blood, urine, and milk samples to study the effects of diseases and toxicants on cattle [12,13,14,15,16].

Conversely, techniques such as mass spectrometry combined with gas chromatography (GC/MS) [17,18], liquid chromatography [19,20], or capillary electrophoresis [21] have high specificity and sensitivity. GC/MS is potentially useful for metabolomics, because of its high sensitivity, peak resolution, and reproducibility [22]. However, for GC/MS analysis, compounds must be volatile and thermally stable; therefore, because most metabolites are polar and nonvolatile, they cannot be readily analyzed by GC/MS [22,23]. Thus, metabolite profiling using GC/MS usually requires chemical derivatization of the polar functional groups of analytes to reduce their polarity and increase their thermal stability and volatility. To our knowledge, only a few studies have evaluated the effects of mycotoxin contamination through GC/MS analysis of naturally contaminated cattle herds. Thus, the main purpose of the present study was to investigate the metabolic effects of mycotoxin contamination in cattle feed under field conditions, especially with regard to ZEN and STC contamination.

## 2. Results

### 2.1. GC/MS Analysis of Urine Samples Derived from Cattle Herds

#### 2.1.1. Original Chromatograms

In the present study, we used GC/MS to identify 55 non-targeted endogenous metabolites related to the metabolism of ATP generation, amino acids, thyroid hormones, neurotransmitters, glycine-conjugation, organic acids, and purine bases, as well as dietary plant components. All identified data was analyzed by ANOVA and/or principal component analysis (PCA) to determine the effects of dietary mycotoxin contamination.

#### 2.1.2. Pattern Recognition

**Figure 1:**
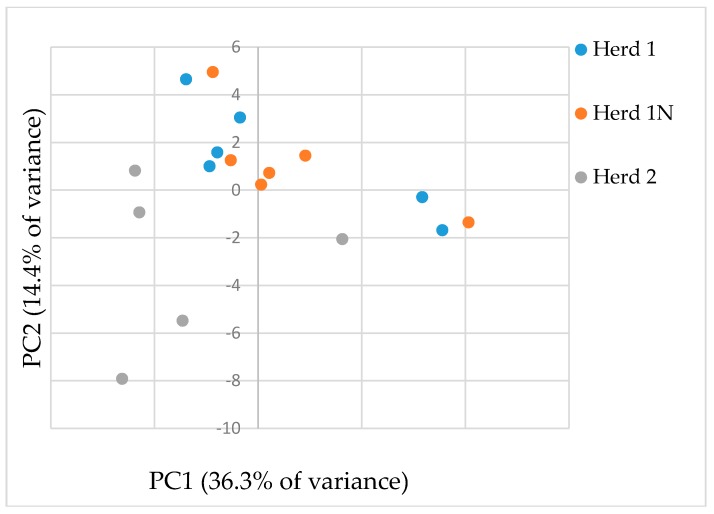
Principal component analysis (PCA) score plots of PC1 versus PC2 from target metabolite profiles of urinary samples derived from three groups of cattle. Herd 1: known to have persistently high urinary ZEN and STC concentrations due to contaminated rice straw, Herd 1N: urine samples were collected from Herd 1, 2 weeks after replacing the newly not-contaminated rice straw, Herd 2: same feeding pattern as Herd 1, except for the contaminated rice straw. The percentages shown on the x- and y-axes represent the contribution to PC1 and PC2, respectively.

Unfortunately, metabolic data from one sample of Herd 2 were not obtained because the derivatization step failed; therefore, data for this sample were not included in the PCA analysis. In the PCA analysis shown in Figure 1, the score plots of the first two principal components (PC1 and PC2) allowed us to visualize and compare the data for the three examined cattle herds.

Data for both Herd 1 and Herd 1N clustered on the upper left-to-right of the plot, and those for Herd 2 fell in the lower left of the plot (PC1 accounted for 36.3% of the variance and PC2 accounted for 14.4%). These results indicated that the metabolite profiles of Herd 1, 1N, and 2 clearly differed. Therefore, based on these results, clustering along PC1 represented the effects of mycotoxin (ZEN and STC) contamination intensity within the dietary feeds, and PC2 represents the effects common to the mycotoxin contamination group. The component loadings that significantly contributed to the clustering of each group along PC1 and/or PC2 are listed in Table 1.

**Figure 2:**
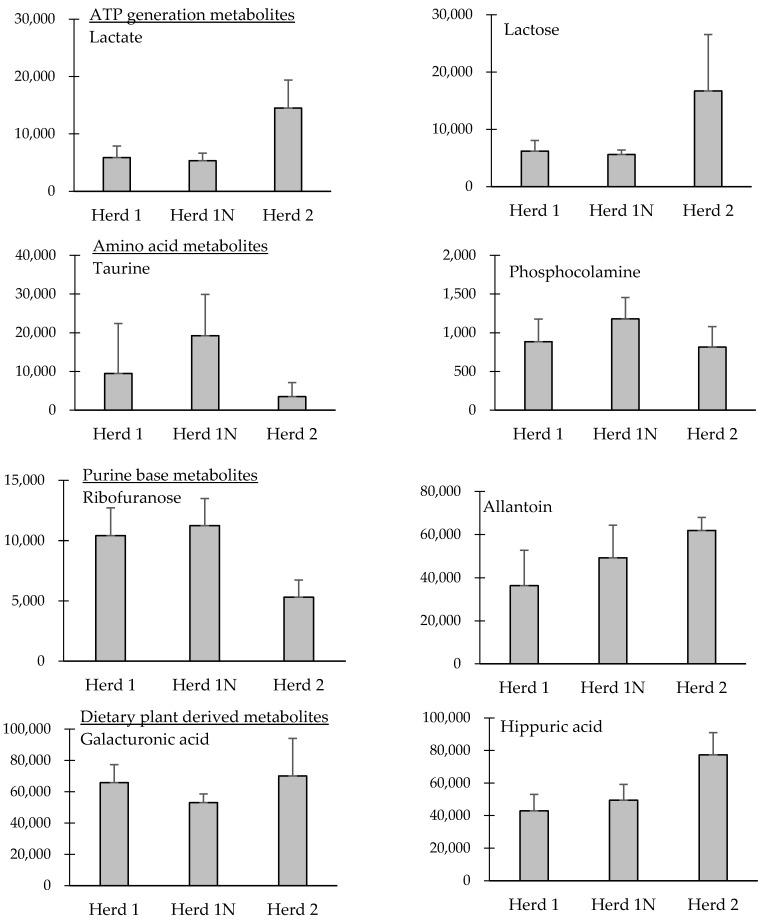
Differences in each representative metabolite among the three herds, which were identified as significantly different by ANOVA (*p* < 0.05). Values on the *y*-axis were normalized to the peak area on the gas chromatography/mass spectroscopy chromatogram by the peak area of internal standard and then normalized by the measured values of urinary creatinine in individual cattle.

Metabolites associated with ATP generation metabolism (xylitol, pantothenic acid, galactose, myo-inositol, aconitic acid, glucose, isocitric acid, lactate, lactose, lyxose, and xylitol), organic acid (hydroxyisovaleric acid, methyl succinic acid, ethylhydracrylic acid, and phenaceturic acid), amino acids (threonine, lysine, taurine, tyrosine, oxoproline, tryptophan, phosphocolamine, and serine), glycine-conjugates (butyrylglycine, hippurate, and methylbutyrylglycine), purine bases (ribofuranose, allantoin, β-pseudouridine, and uric acid), and dietary plant-derived metabolites (adonitol, threitol, cinnamate, hydroxyphenyllactate, glycolic acid, indol-3-acetic acid, galacturonic acid, hippuric acid, deoxytetronic acid, threonic acid, and gluconic acid) significantly contributed to different clusters for the three cattle herds on PCA (Table 1). We used a one-way ANOVA to determine significant differences in the levels of metabolites (creatinine modified area) among the three cattle herds. Results of the ANOVA indicated that there were significant differences in the amounts of 11 metabolites among the three herds, as shown in Table 1 and Figure 2.

## 3. Discussion

Metabolomics can be efficiently utilized to clarify several simultaneous effects in a single examination, and can identify temporal variation in the metabolic responses of living organisms to physiological and pathological stress conditions [24,25,26]. Although several studies have investigated the toxicity of acute exposure to ZEN or other kinds of mycotoxins in laboratory animals, only a few have obtained data from cattle urine samples, from which chronic contamination with ZEN and STC were identified. To our knowledge, the present metabolomics study reports, for the first time, the chronic effects of mycotoxin contamination on cattle metabolism.

Previous studies investigating the effects of ZEN have involved measuring and comparing levels of a single or several biochemical markers. Seeling et al. [27] reported that the effects of Fusarium toxin intake at 8.21 mg DON/kg DM and 0.09 mg ZEN/kg DM were insufficient to induce any toxicological changes in serum biochemical parameters of protein metabolism or liver damage, such as serum aspartate aminotransferase (AST), γ-glutamyltransferase (GGT), total protein (TP), or serum albumin (Alb) in cows. Additionally, we previously investigated serum samples from animals of Herds 1, 2, and 1N, and no significant differences were observed in protein metabolism (TP, Alb, albumin/globulin ratio, and blood urea nitrogen), liver function indicators (AST, GGT, and insulin like growth factor-1), or energy metabolism (total cholesterol and glucose), except for free fatty acids, which may be indicative of an energy imbalance [3]. However, based on a recent consensus regarding the evaluation of adverse effects of mycotoxins, traditional methods may not be sensitive enough to determine the systemic metabolic status of animals [2,24].

Previously, ZEN exposure was shown to induce oxidative stress, and some common changes in systemic metabolism, including effects on cell membrane metabolism, protein biosynthesis, glycolysis, and gut microbiota metabolism in rodents [2]. Additionally, Santos and Fink-Gremmels [28] performed genomic analyses in dairy cattle and reported that dysfunctional lipid metabolism and oxidative stress seemed to be key elements of the mycotoxin syndrome. Metabolomics studies with experimental animals have also shown that higher concentrations of metabolites in rat urine samples indicate high levels of metabolic waste or the ineffective use of nutrients, which are subsequently excreted [16]. Therefore, in the present study, we evaluated the metabolic status of animals considering the findings of previous studies as well as the general consensus.

### 3.1. Metabolism of ATP Generation

In the present study, seven detected metabolites were associated with gluconeogenesis, and significantly/strongly contributed to the separation along PC1 (positively), and four detected metabolites were along PC2 (negatively) in the PCA. Additionally, significant differences were observed for both urinary lactate and lactose among the three groups by ANOVA analysis. Our results indicate that mycotoxin exposure may affect gluconeogenesis/energy metabolism in cattle, as reported in experimental animals [2]. Xylitol is produced by the hydrogenation of xylose derived from hemicellulose, which is one of the main constituents of biomass, and can be extracted from the fibrous material of oats, cornhusks, and sugar cane bagasse [29]. Carbohydrates in dietary feed are ultimately digested as glucose, galactose, and fructose, which are absorbed from intestinal epithelial cells and utilized for gluconeogenesis in the liver at the end of several metabolic pathways. Lactose is a disaccharide derived from galactose and glucose, and is usually found in milk. In the present study, the levels of urinary xylitol, galactose, and glucose in the contaminated herds were significantly higher than those in the control herd; the inverse was found for urinary lactose and lactate, which were significantly higher in the control herd than in the contaminated herds. Lactate is the end-product of energy metabolism/glycolysis [2]. Previous studies involving experimental animals have indicated that the concentration of plasma lactate increases following ZEN exposure [2,30]. Gluconeogenesis has been shown to occur during long-term starvation; however, similar metabolic changes can occur when organisms encounter harmful stimuli [31]. Additionally, previous studies on experimental animals have suggested that increased glucose utilization might be a major metabolic effect of mycotoxin exposure [2,32]. We previously reported that there was no difference in the serum glucose concentration between Herds 1 and 2 at the time of urine collection (74.8 ± 0.9 and 65.4 ± 3.5 mg/dL, respectively) [3]. Therefore, our results may indicate that increased gluconeogenesis and reduced glycolysis in animals of Herds 1 and 1N are similar as they attempt to maintain glucose concentration.

Based on the results of the PCA, urinary pantothenic acid, myo-inositol, aconitic acid, and isocitric acid were found to differ significantly between contaminated and control herds. Animals require pantothenic acid to synthesize coenzyme-A (CoA) [33]. CoA is important in energy metabolism, and is needed for pyruvate to enter the tricarboxylic acid cycle (TCA cycle). Myo-inositol is an important structural molecule for a number of secondary messengers in eukaryotic cells, including insulin signal transduction, cytoskeleton assembly, control of intercellular calcium concentration, and the breakdown of fats [34].

In the present study, a significant difference in methyl succinic acid, hydroxyisovaleric acid (HIVA), and uric acid was observed between the contaminated and control groups. The formation of 3-HIVA in mammals has been demonstrated in association with leucine degradation, and is normally present in small quantities in urine [35]. In a previous study, increased concentrations of 3-HIVA were found in all urine samples from patients with ketoacidosis [35]. This observation may suggest a mild ketotic condition in contaminated herds, even without any difference in serum glucose concentrations between contaminated and control herds. Analysis of beta-hydroxybutyrate must be performed in the future to clarify this point.

### 3.2. Amino Acid Metabolism

In the present study, significant differences in the essential amino acids, threonine, and lysine, and taurine were observed, resulting in the separation of contaminated and control groups along with PC1 in PCA. These results suggest that mycotoxin exposure alters amino acid metabolism in cattle. Threonine is converted to pyruvate via threonine dehydrogenase, and an intermediate in this pathway can undergo thiolysis with CoA to produce acetyl-CoA and glycine. Lysine is metabolized in mammals to produce acetyl-CoA, via initial transamination with α-ketoglutarate. Because both threonine and lysine are essential amino acids, they must be obtained from the diet, or are biosynthesized within the rumen in cattle. As the diet composition of the individual herds was almost the same, except for the contaminated rice straw, variation can be attributed to the different levels of mycotoxin exposure. Previous studies have shown that ZEN can inhibit protein synthesis in experimental animals [2,36], which might be due to stress-induced increases in energy expenditure, and thus the elevated consumption of amino acids. However, in the present study, urinary extraction was increased upon feeding with mycotoxin-contaminated diets, especially for taurine. Our results seem to contradict the results of other studies with experimental animals, but the causes of these phenomena are obscure. Another approach may be necessary to clarify the differences.

In the present study, levels of tyrosine and oxoproline were significantly different between contaminated and control herds. Notably, tyrosine is an important amino acid in many proteins, peptides, and even enkephalins, and is the precursor of thyroid hormones [37]. Oxoproline is a metabolite involved in the glutathione cycle, which is converted to glutamate. The liver represents an important pool for amino acid metabolism, and is vital for the decomposition and utilization of most amino acids in cattle, except for branched-chain amino acids [38]. Our results suggest that excessive protein synthesis occurs to maintain the concentration of thyroid hormone.

In the present study, levels of tryptophan, phosphocolamine, and serine significantly differed between the contaminated and control herds along PC1 of the PCA. Additionally, a significant difference was observed in phosphocolamine among the three herds. Tryptophan is a precursor of the neurotransmitters serotonin and melatonin. Phosphocolamine is an ethanolamine derivative that is used to construct two different types of phospholipids; glycerophospholipid and sphingomyelin. Serine, a non-essential amino acid, is important in metabolism, and participates in the biosynthesis of purines and pyrimidines. In addition, it is the precursor of several amino acids, including glycine, cysteine, and tryptophan in bacteria, and plays an important role in the catalytic function of many enzymes. 5-hydroxytryptamine is a messenger generated from tryptophan, which can produce pleasurable emotions and affects nearly all types of brain activity [39]. Previous studies have shown that low levels of 5-hydroxytryptamine can lead to depression [40]. Thus, our results indicate that increased levels of neurotransmitter-related amino acids may explain the inappetence and depression observed in cattle exposed to ZEN and STC mycotoxin contamination.

### 3.3. Glycinconjugation

In the present study, three metabolites associated with glycine conjugates, hippurate, butyrylglycine, and methylbutyrylglycine, significantly contributed to the separation of contaminated and control herds along with PC1. The results of a previous study suggested that both hippurate and glycine conjugates are normally present in the urine of healthy cattle, and that the increased urinary excretion of glycine conjugates is related to detoxification [12]. Hippurate is a normal component in urine and is formed by the conjugation of benzoic acid and glycine; it is thought that the amount of urinary hippurate may reflect the hepatic detoxification ability by means of glycine conjugation [12]. Butyrylglycine and methylbutyrylglycine are acyl glycines, which are normally minor metabolites of fatty acids. In some cases, the levels of these metabolites in body fluids can be used to diagnose disorders associated with mitochondrial fatty acid beta-oxidation. The metabolism of xenobiotics is often divided into three phases: Phase I, modification; Phase II, conjugation; and Phase III, excretion. In subsequent Phase II reactions, these activated xenobiotic metabolites are conjugated with charged species such as glutathione, sulfate, glucuronic acid, and glycine. Therefore, the results of the present study suggest that mycotoxin contamination (ZEN and STC exposure) in cattle may have affected detoxification during Phase II.

### 3.4. Purine Base Metabolism

Urine metabolites, such as purine derivatives, have been used to estimate levels of microbial protein synthesis in the rumen [41]. In the present study, levels of ribofuranose, allantoin, β-pseudouridine, and uric acid differed significantly between contaminated and control herds. Additionally, significant differences were observed in ribofuranose among the three herds. Ribofuranose forms part of the RNA backbone, which is related to deoxyribose. Uric acid is a bi-product of the metabolic breakdown of purine nucleotides, and in most mammals, the enzyme uricase further oxidizes uric acid to allantoin. Hypouricemia may occur following exposure to drugs and toxic agents [42]. The presence of allantoin in the urine can occur via non-enzymatic means, through high levels of reactive oxygen species; thus, allantoin is thought to be a marker of oxidative stress [2]. A previous report indicated that serological parameters of oxidative stress are the most practical parameters associated with dietary multiple mycotoxin contamination in dairy cattle [28]. Allantoin is a product of purine metabolism, and a strong correlation between the urinary excretion of purine metabolites and allantoin has been reported [43]. Urinary purine metabolite excretion seems to be an indicator of microbial protein synthesis in ruminants [44,45]. Additionally, Sun et al. [16] reported a negative relationship between allantoin and nitrogen efficiency. Therefore, our results suggest that mycotoxin (ZEN and STC) exposure may oxidatively affect stress as well as nitrogen efficiency in the rumen of cattle.

### 3.5. Metabolites Possibly Derived from Dietary Components

In the present study, the levels of nine identified metabolites associated with feed materials differed significantly between contaminated and control herds, including adonitol, threitol, cinnamate, hydroxyphenyllactate, glycolic acid, indol-3-acetic acid, deoxytetronic acid, threonic acid, and gluconic acid. Additionally, significant differences were observed in the levels of galacturonic acid, hippuric acid, and glycolic acid among the three herds. Adonitol is a crystalline pentose alcohol formed via the reduction of ribose, which occurs in the cell walls of Gram-positive bacteria. Cinnamate is found naturally in a variety of plants. It is thought that hydroxyphenyllactate is derived from roughage. Glycolic acid is found in some sugar-crops. Indol-3-acetic acid is the most common naturally occurring plant hormone of the auxin class. Therefore, the above results may reflect the difference in dry matter content in dietary feed between the contaminated and control herds, and the subsequent nutrient utilization by the animals. Conversely, galacturonic acid is a sugar acid- oxidized form of galactose, and is the main component of pectin. Pectin consists of a complex set of polysaccharides, which are present in most primary cell walls of plants. As noted, because urinary galactose excretion contributed to the separation of contaminated and control herds, this may affect/reflect the significant differences in urinary galacturonic acid excretion observed in the present study. Hippuric acid is a carboxylic acid found in the urine of horses and other herbivores. Although rumen microorganisms may synthesize benzoic acid from a variety of sources, benzoic acid is not normally synthesized by animals, and ingested benzoic acid is excreted as hippuric acid in the urine of herbivorous animals following conjugation with glycine [46]. Hippuric acid in bovine urine is strongly associated with its concentration in dietary feed [12]. Therefore, the different levels of urinary hippuric acid observed between the contaminated and control herds may reflect its volume in the straw feed.

## 4. Conclusions

In conclusion, the results of the present study indicate that the contamination of cattle feed with multiple mycotoxins, as confirmed by the measurement of ZEN and STC, may result in altered metabolic processes, including ATP generation, amino acid, and purine base metabolism. The results obtained from Herd 1N indicate that the two-week remedy period for contaminated feed was not sufficient to modify most of the urinary metabolites, suggesting that chronic contamination of mycotoxins may have long-term adverse effects on systemic metabolism in cattle. This suggests that monitoring endogenous metabolites in urine samples (metabolomics) is useful for evaluating the effects of cattle exposure to multiple mycotoxins. Further field studies are required to develop a basic tool to evaluate the impact of chronic exposure to low-dose mycotoxins on cattle health and productivity. Additionally, because of the limitations of using GC/MS as the analytical tool for metabolomics analysis, extensive sample treatment and analytical validation must be performed.

## 5. Materials and Methods

Animals were cared for according to the Guide for the Care and Use of Laboratory Animals (Joint Faculty of Veterinary Medicine, Yamaguchi University, Yamaguchi, Japan).

### 5.1. Chemicals and Reagents

*N*-methyl-*N*-(trimethylsilyl)-trifluoroacetamide with 1% trimethylchlorosilane (MSTFA + 1% TMCS) was purchased from Thermo Scientific (Pittsburgh, PA, USA). *O*-methylhydroxylammonium chloride (methoxylamine hydrochloride), pyridine, 2,2-dimethyl succinic acid, myristic acid d27, pesticide-analytical-grade chloroform and hexane, and high-performance liquid chromatography (HPLC)-analytical-grade methanol were purchased from Wako Pure Chemical (Tokyo, Japan). Urease was purchased from Sigma-Aldrich (St. Louis, MO, USA).

### 5.2. Sample Collection and Processing

Two herds of female Japanese Black cattle (Herds 1 and 2; 23-month old, weighing 550–600 kg) maintained for fattening for meat in Kagoshima Prefecture, Japan, were used in this study. Herd 1 is known to have persistently high urinary ZEN and STC concentrations due to the contamination of rice straw. Herd 2 had the same dietary feed as Herd 1, but were fed with rice straw not contaminated with ZEA and STC. The content of the feed given to each herd is described in Table 2. Urine samples were collected from individual animals (*n* = 6 per herd) 2 h after the morning feed by massaging the perineum. Additional urine samples were collected from Herd 1, 2 weeks after replacing their rations with newly un-contaminated rice straw (Herd 1N). ZEN and STC concentrations in the dietary straw, ZEN, and the concentrations of its metabolites; α-Zearalenol (α-ZOL), and β-Zearalenol (β-ZOL), in urine samples of cattle derived from each experimental group collected on the same day, including the results of the dietary straw and urine samples collected at 42 or 34 days before (for Herds 1 and 2, respectively), have been partially reported previously (Hasunuma et al., 2012; Fushimi et al., 2014a, b), and are shown in Table 3. These results indicate that the administration of the contaminated rice straw in Herd 1 lasted at least for 42 days. During the 2-week period, no clinical differences were observed, except for the physical appearance of the buttocks which may reflect the fecal discharge condition. All the samples were immediately placed in a cool box, protected from light, and were transported to the laboratory. Urine samples were centrifuged (Model 2410, Kubota Corp., Tokyo, Japan) at 500× *g* for 10 min at room temperature to remove debris and frozen at −30 °C until GC/MS analysis.

### 5.3. Preparation of Urine Samples for GC/MS Analyses

After thawing at room temperature, the urine samples were centrifuged (Model 6000, Kubota Corp., Tokyo, Japan) at 10,000 rpm for 10 min at 4 °C. Aliquots of urine samples (100 μL) were transferred into 2-mL polypropylene (PP) microtubes, and 100 μL urease (2 mg/mL) was added. Samples were incubated for 30 min at 37 °C with shaking. One-milliliter of methanol cooled with ice was added to the urine samples, which were then shaken on a vortex mixer, and centrifuged at 10,000 rpm for 10 min at 4 °C. Next, 900 μL of the upper layer was placed in another 2-mL PP microtube, spiked with 2,2-dimethyl succinic acid and myristic acid d27 for use as the internal standards, and then evaporated to dryness under a gentle stream of nitrogen at room temperature. Derivatization was performed in two steps: oximation and silylation. To this end, the dried residue was dissolved in 10 μL of a pyridine solution of methoxylamine hydrochloride (40 mg/mL) by means of continuous shaking at 30 °C for 90 min, then silylation was carried out with 90 μL of MSTFA + 1% TMCS at 37 °C for 30 min. After derivatization, 100 μL of hexane was added to each sample, which were additionally diluted 10 times with hexane, and rapidly injected into the GC/MS.

### 5.4. GC/MS Analysis and Creatinine Levels in the Urine

Metabolites were analyzed on an Agilent Technologies 6890 Series gas chromatograph equipped with a 5973 MSD mass selective detector and a DB 5-ms capillary column (i.d. 0.25 mm × 30 m, 0.25-μm film thickness; J&W Scientific, Folsom, CA, USA). The injected sample volume was 1 μL. The injector and detector temperatures were 250 °C and 290 °C, respectively; the oven temperature program was as follows: 60 °C for 1 min, increased to 325 °C at 10 °C/min, and then held at 325 °C for 12 min. In the present study, we replicated the GC/MS analysis with the same urine to avoid experimental fluctuations and misinterpretations.

Creatinine concentrations in the urine were determined using a commercial kit (Sikarikit-S CRE; Kanto Chemical, Tokyo, Japan) according to the manufacturer’s instructions and measured using a clinical autoanalyzer (7700 Clonical Analyzer; Hitachi High-Tech, Tokyo, Japan).

### 5.5. Data Processing

Metabolite peaks on the GC/MS chromatogram were drawn from the baseline to obtain the peak area, deconvoluted, and aligned using MetAlignTM (ver. 080311, Wageningen University, Wageningen, The Netherlands). Data obtained from peak areas for individual target metabolites and normalized by the peak area of 2,2-dimethyl succinic acid as an internal standard were additionally normalized by the creatinine levels measured in individual cattle. The normalized values were analyzed by one-way analysis of variance (ANOVA). Then PCAs were performed for the metabolites that differed significantly by ANOVA among all groups, in order to evaluate differences in toxic effects among the groups. PCA score plots were used with component loading to evaluate how specific metabolites were affected by exposure to mycotoxins. Statistical analyses were performed with the R programming language (http://www.r-project.org/). *p* < 0.05 was considered significant.

## Figures and Tables

**Table 1:** Metabolites contributing to the clustering of each group along PC1 and PC2 on the PCA score plot, and *p* values showing significant differences by one-way ANOVA among the three cattle herds.

	Metabolite	PC1	PC2	*p* Value
ATP generation	xylitol	0.88		0.12
	pantothenic acid	0.79		0.63
	galactose	0.59		0.79
	myo-inositol	0.56		0.51
	aconitic acid	0.54		0.55
	glucose	0.53		0.78
	isocitric acid	0.53		0.50
	lactate		−0.87	0.038
	lactose		−0.84	0.03
	lyxose		−0.59	0.47
	xylitol		−0.50	0.80
Organic acid	hydroxyisovaleric acid	0.64		0.48
	methyl succinic acid	0.66		0.32
	ethylhydracrylic acid		−0.69	0.19
	phenaceturic acid		−0.63	0.35
Amino acid	threonine	0.78		0.08
	lysine	0.64		0.31
	taurine	0.52		0.028
	tyrosine	0.86		0.36
	oxoproline (pyroglutamic acid)	0.70		0.38
	tryptophan	0.74		0.32
	phosphocolamine	0.73		0.0008
	serine	0.62	−0.61	0.09
Glycin-conjugate	butyrylglycine	0.76		0.70
	hippurate	0.75		0.66
	methylbutyrylglycine	0.62		0.82
Purine base	ribofuranose	0.76		0.003
	allantoin		−0.56	0.011
	β-pseudouridine	0.85		0.87
	uric acid	−0.60	−0.58	0.23
Dietary plant-derived	adonitol	0.88		0.87
	threitol	0.88		0.87
	cinnamate	0.82		0.30
	hydroxyphenyllactate	0.82		0.48
	glycolic acid	0.56	−0.48	0.75
	indol-3-acetic acid	0.54		0.54
	galacturonic acid		−0.66	0.002
	hippuric acid		−0.67	0.0002
	deoxytetronic acid	0.95		0.53
	threonic acid	0.90		0.68
	gluconic acid	0.88		0.77

**Table 2:** Composition of feed provided to the 2 cattle herds kept for fattening purpose (as-fed basis).

	Forage Feed, kg	Formula Feed						
Herd		Total, kg	Bran, %	Cereal, %	Oil Seed Meal, %	Other, %	TDN, %	CP, %
Herd 1	Straw, 2	9	17	77	5	1	>75	>14
Herd 2	Straw, 4	9	17	77	5	1	>75	>14

These results have been reported previously (Hasunuma et al., 2012).

**Table 3:** Zearalenone (ZEN) and sterigmatocystin (STC) concentrations in the dietary straw, and ZEN, α-Zearalenol (α-ZOL), and β-Zearalenol (β-ZOL) concentrations in urine samples of cattle derived from each experimental group collected on the same day.

Experimental Group	Straw (mg/kg)		Urine Samples (Mean ± SEM; pg/mg Creatinine)			
	ZEN	STC	ZEN (*n* = 6)	α-ZOL (*n* = 6)	β-ZOL (*n* = 6)	STC (*n* = 6)
Herd 1	7.6	0.24	3702 ± 747	859 ± 178	5503 ± 1130	569 ± 111
(−42 days) *	7.5	0.17	2444 ± 394	759 ± 148	5496 ± 1197	209 ± 22
Herd 1N	ND	0.04	63 ± 12	20 ± 3	119 ± 18	33 ± 8
Herd 2	0.2	<0.01	50 ± 8	ND	82 ± 27	47 ± 19
(−34 days) **	0.2	0.03	68 ± 10	21 ± 9	215 ± 51	147 ± 39

These concentrations in straw and urine samples have been partially reported previously (Hasunuma et al., 2012 and Fushimi et al., 2014a, b). *: Straw and urine samples collected 42 days before the sampling for metabolomics in Herd 1. **: Straw and urine samples collected 34 days before the sampling for metabolomics in Herd 2.

## Supplementary Materials

The following are available online at www.mdpi.com/2072-6651/9/10/294/s1, Table S1: All detected/identified 55 analytes, Figure S1: Representative chromatogram of the GC/MS analysis in the present study.
