# Supplementary material for: Gas Chromatography-Mass Spectrometry for Metabolite Profiling of Japanese Black Cattle Naturally Contaminated with Zearalenone and Sterigmatocystin
Source: Toxins (Basel). 2017 Sep 21;9(10):294. doi: 10.3390/toxins9100294 (PMC5666341; doi:10.3390/toxins9100294)
Supplement: Supplementary file 1 [file toxins-09-00294-s001.pdf]

Supplementary Materials: Gas Chromatography-Mass Spectrometry for Metabolite Profiling of Japanese Black Cattle Naturally Contaminated with Zearalenone and Sterigmatocystin

Katsuki Toda, Emiko Kokush, Seiichi Uno, Ayaka Shiiba, Hiroshi Hasunuma, Yasuo Fushimi, Missaka P.B. Wijayagunawardane, Chunhua Zhang, Osamu Yamato, Masayasu Taniguchi, Johanna Fink-Gremmels and Mitsuhiro Takagi

Table S1. All detected/identified 55 analytes; retention time (RT), derivation, quantifire ion (m/z) and qualifirier ion (m/z).

Metabolites (Derivatized by TMS)	RT	Quantifire Ion (m/z)	Qualifirier Ion (m/z)
Lactate	6.27	117	147
Glycolic acid	6.52	147	73
Alanine	6.93	116	147
Hydroxyisovaleric acid	8.52	147	117
Isovaleric acid	8.52	131	147
Ethylhydracrylic acid	8.79	147	247
Ethanolamine	8.84	174	100
Succinic acid	10.00	247	147
Glycine	10.02	174	248
2,2-Dimethyl sccunic acid	10.09	231	275
Methyl succinic acid	10.15	261	147, 217
Gryceric acid	10.20	292	189
Deoxytetronic acid	10.37	292	147
Serine	10.61	204	218
Threonine	10.94	218	219, 291
Methylcatechol	11.02	268	73
Deoxytetronic acid	11.29	103	219
β-Alanine	11.52	248	174, 290
Methylbutyrylglycine	12.10	172	216, 203
Butyrylglycine	12.21	172	274, 102
Threitol	12.51	217	307, 205
Oxoproline (Pyroglutamic acid)	12.69	156	230
Threonic acid	12.94	292	205, 220
Creatinine	13.11	314	329
Hydroxyphenylacetic acid	14.12	296	296
Lyxose	14.26	307	217
Taurine	14.42	326	269
Xylitol	14.84	217	307, 319
Adonitol	15.05	217	307, 319
Acconitic acid	15.32	229	147
Glycerol-3-phosphate	15.44	357	299, 455
Phosphocolamine	15.68	174	188
Myristic-*d27* acid (internal standard)	16.21	73	147
Hippuric acid	16.37	206	105

Phenaceturic acid	16.72	218	147
Allantoin	16.79	331	431, 446
Hydroxyphenyllactate	16.91	179	308
Lysine	16.91	174	317
Galacturonic acid	17.26	333	160
Tyrosine	17.32	218	280
Gluconic acid	17.55	333	292
Pantothenic acid	17.82	219	420
Glucuronic acid	18.06	217	204
myo-Inositol	18.77	205	318
Uric acid	18.84	441	456
Glucose	18.95	217	204
Cinnamate	19.14	319	205
Galactose	19.19	319	205
Indol-3-acetic acid	19.54	290	407
Tryptophan	19.71	218	130
Pseudouridine	20.9	357	217
Galactopyranoside	21.07	204	337
Ribofuranose	21.42	217	147
p-Tolyl- β -D-glucuronide	21.67	375	180
Monopalmitin	22.95	371	459
Mannobiose	23.7	301	204
Lactose	23.82	319	204

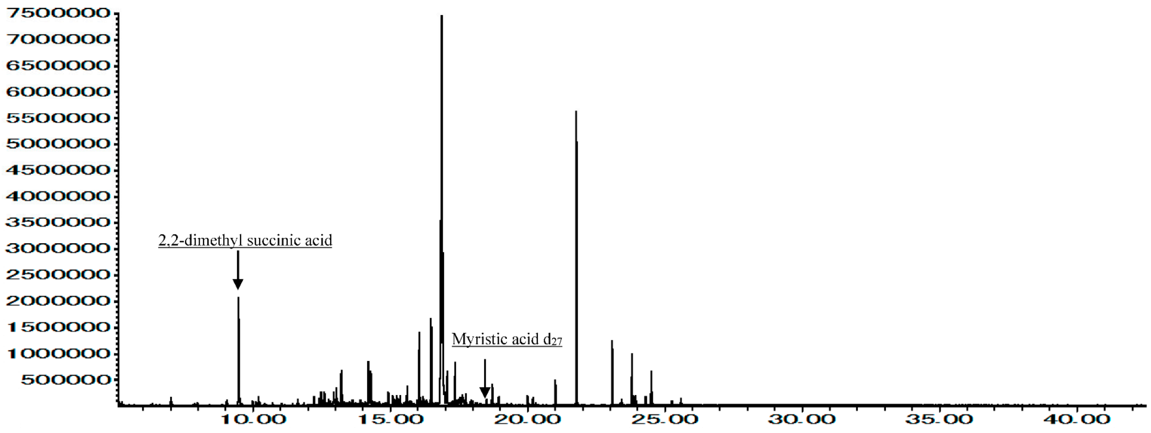

Figure S1. Representative chromatogram of the GC/MS analysis in the present study.
